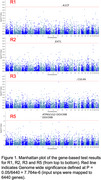# Genomic and clinical correlates of 5 deep learning‐based neuroimaging signatures of neurodegeneration, evaluated in ADSP

**DOI:** 10.1002/alz70855_101581

**Published:** 2025-12-23

**Authors:** Yuhan Cui, Dhivya Srinivasan, Zhijian Yang, Junhao Wen, Guray Erus, Timothy J. Hohman, Andrew J. Saykin, Paul M. Thompson, Li Shen, Christos Davatzikos

**Affiliations:** ^1^ Artificial Intelligence in Biomedical Imaging Laboratory, Perelman School of Medicine, University of Pennsylvania, Philadelphia, PA, USA; ^2^ Center for Innovation in Imaging Biomarkers and Integrated Diagnostics (CIMBID), Department of Radiology, Columbia University, New York, NY, USA; ^3^ Columbia University, New York, NY, USA; ^4^ Vanderbilt Memory & Alzheimer's Center, Vanderbilt University Medical Center, Nashville, TN, USA; ^5^ Center for Neuroimaging, Department of Radiology and Imaging Sciences, Indiana University School of Medicine, Indianapolis, IN, USA; ^6^ Imaging Genetics Center, Mark and Mary Stevens Neuroimaging & Informatics Institute, University of Southern California, Marina del Rey, CA, USA; ^7^ Department of Biostatistics, Epidemiology, & Informatics, University of Pennsylvania, Philadelphia, PA, USA; ^8^ Center for AI and Data Science for Integrated Diagnostics, University of Pennsylvania, Philadelphia, PA, USA

## Abstract

**Background:**

Deep learning methods help to disentangle heterogeneity of brain aging and find distinct neuroimaging patterns of neurodegeneration. However, the relationship between aging‐related brain atrophy patterns and genetics is complex and requires further exploration.

**Method:**

Patterns of regional bran volumes extracted from T1 scans and Whole‐genome sequencing (WGS) data in The Alzheimer's Disease Sequencing Project (ADSP) were analyzed (2401 subjects, age = 72.75 ± 9.08; 54.07% female). We investigated 5 recently published brain aging patterns (R‐indices) (Yang et al., 2024), and evaluated out‐of‐sample reproducibility of previously reported associations between R‐indices and diagnostic groups (CN vs MCI/AD). We conducted genome‐wide association analysis to investigate genetic associations of R‐indices controlling for confounders (e.g., age).

**Result:**

Linear regression models identified significant group differences with large effect size in diagnostic groups in R2 (Cohen's d=1.05, *p* = 1.66e‐105; this is a typical AD pattern of atrophy) and R3 (d=0.81, *p* = 2.16e‐66), and moderate effect size in R5 (d=0.44, *p* = 9.59e‐22; this pattern was previously associated with a variety of cardiovascular risk factors and immune system markers). Genetic analyses identified 5 genes significantly associated with R1, R2, R3 and R5 indices (Figure 1). R1, characterized with subcortical atrophy, had associations with A1CF (chr10), which has been associated with urate levels, gout and colorectal cancer. R2, with focal medial temporal lobe atrophy, was associated with EXT1 (chr8), which has been associated with BMI, general cognitive ability, cortical thickness and insomnia. R3, with parieto‐temporal atrophy patterns, was associated with CUL4A (chr13), which has been linked to blood cell volume/distribution, bipolar disorder, HDL cholesterol, corpuscular hemoglobin and atrial fibrillation. R5, with primarily perisylvian atrophy, was associated with DDX39B and ATP6V1G2‐DDX39B (chr6), which have been associated with insomnia, general cognitive ability and blood cell measures.

**Conclusion:**

We evaluated 5 recently established brain aging indices, which capture the heterogeneity of structural brain aging, in ADSP participants. R‐indices replicated previously reported associations with AD groups (Yang et al., 2024), indicating the generalizability of the model. We identified different associations with genes that were previously linked to various traits. These findings provide new insights into the exploration of heterogeneity of neurodegeneration and related genetic risk factors.